# 

**Published:** 1883-10-20

**Authors:** 


**NOTICE.**—*Many of our subscribers have forgotten us in the matter of remitting their dues. Friends, please attend to this matter at once. We are obliged to have money to run the Journal. Our printers are Cash men.* W.

### *EDITORIAL NOTICES.*

PARKE, DAVIS & CO., the great Drug house of Detroit, have a new and interesting advertisement in this issue.

SADDLE-BAGS.—See the new advertisement of A. A. Mellier, of St. Louis, in this number. His Saddle-bags can't be beat.

McKESSON & ROBBINS.—We ask attention to the new advertisement of the above excellent house, commencing in this number of our Journal.

FRANKLIN MILLS CO.—See the advertisement of Warren's Food Flour, containing the entire elements of the wheat. Let our friends test this article of flour, especially in dyspeptic cases.

W. S. MERRELL CHEMICAL CO.—Examine the advertisement of this large and popular house, Cincinnati, Ohio, successors to the late Wm. S. Merrell & Co., well and favorably known as Manufacturing Chemists in that city.

Cumming Clarion: The following placard is to be seen on the wall of a country store in this county:

PEPPERMINT ILE FOR

Hed ake,
Belle "
Toth "

### *RECEIPTED.*

1883—Drs. Robert M Savage, M E Demaret, W P Watson, to August; G W Earl, to August; G L Danders, to November; C F Yeager, T B Meacham, J R Burton, J W Mitchell, N P Shelly, J R Muse, L M Lovelace, '82; W Barton, '84; J W Burnett, '84; C H Chalkly; to July '84; Wm R Smith, '84; Thomas N Clement, '84; James Warren, '84; R C Norwood, 1884.

